# A recombinant plasmid encoding human hepatocyte growth factor promotes healing of combined radiation-trauma skin injury involved in regulating Nrf2 pathway in mice

**DOI:** 10.1093/jrr/rrae011

**Published:** 2024-04-28

**Authors:** Dujuan Li, Yuxin Lu, Fengjun Xiao, Xiaochen Cheng, Chunsheng Hu, Xuefeng Zhu, Xiaoying Wang, Haiying Duan, Li Du, Qinglin Zhang

**Affiliations:** Department of Pharmacy & Pharmacology, University of South China, 28 Changsheng West Road, Zhengxiang District, Hengyang, Hunan 421001, China; Department of Experimental Hematology and Biochemistry, Beijing Institute of Radiation Medicine, 27 Taiping Road, Haidian District, Beijing 100850, China; Department of Experimental Hematology and Biochemistry, Beijing Institute of Radiation Medicine, 27 Taiping Road, Haidian District, Beijing 100850, China; Department of Experimental Hematology and Biochemistry, Beijing Institute of Radiation Medicine, 27 Taiping Road, Haidian District, Beijing 100850, China; Department of Experimental Hematology and Biochemistry, Beijing Institute of Radiation Medicine, 27 Taiping Road, Haidian District, Beijing 100850, China; Department of Pharmacology, College of Pharmacy & International Academy of Targeted Therapeutics and Innovation, National & Local Joint Engineering Research Center of Targeted and Innovative Therapeutics, Chongqing University of Arts and Sciences, 319 Honghe avenue, Yongchuan District, Chongqing 402160, China; Department of Experimental Hematology and Biochemistry, Beijing Institute of Radiation Medicine, 27 Taiping Road, Haidian District, Beijing 100850, China; Department of Experimental Hematology and Biochemistry, Beijing Institute of Radiation Medicine, 27 Taiping Road, Haidian District, Beijing 100850, China; Department of Experimental Hematology and Biochemistry, Beijing Institute of Radiation Medicine, 27 Taiping Road, Haidian District, Beijing 100850, China; Department of Experimental Hematology and Biochemistry, Beijing Institute of Radiation Medicine, 27 Taiping Road, Haidian District, Beijing 100850, China; Department of Experimental Hematology and Biochemistry, Beijing Institute of Radiation Medicine, 27 Taiping Road, Haidian District, Beijing 100850, China

**Keywords:** combined radiation-trauma skin injury, hepatocyte growth factor, Nrf2, plasmid

## Abstract

Combined radiation-trauma skin injury represents a severe and intractable condition that urgently requires effective therapeutic interventions. In this context, hepatocyte growth factor (HGF), a multifunctional growth factor with regulating cell survival, angiogenesis, anti-inflammation and antioxidation, may be valuable for the treatment of combined radiation-trauma injury. This study investigated the protective effects of a recombinant plasmid encoding human HGF (pHGF) on irradiated human immortalized keratinocytes (HaCaT) cells *in vitro*, and its capability to promote the healing of combined radiation-trauma injuries in mice. The pHGF radioprotection on irradiated HaCaT cells *in vitro* was assessed by cell viability, the expression of Nrf2, Bcl-2 and Bax, as well as the secretion of inflammatory cytokines. *In vivo* therapeutic treatment, the irradiated mice with full-thickness skin wounds received pHGF local injection. The injuries were appraised based on relative wound area, pathology, immunohistochemical detection, terminal deoxynucleotidyl transferase dUTP nick end labelling assay and cytokine content. The transfection of pHGF increased the cell viability and Nrf2 expression in irradiated HaCaT cells. pHGF also significantly upregulated Bcl-2 expression, decreased the Bax/Bcl-2 ratio and inhibited the expression of interleukin-1β and tumor necrosis factor-α in irradiated cells. Local pHGF injection *in vivo* caused high HGF protein expression and noticeable accelerated healing of combined radiation-trauma injury. Moreover, pHGF administration upregulated Nrf2, vascular endothelial growth factor, Bcl-2 expression, downregulated Bax expression and mitigated inflammatory response. In conclusion, the protective effect of pHGF may be related to inhibiting apoptosis and inflammation involving by upregulating Nrf2. Local pHGF injection distinctly promoted the healing of combined radiation-trauma injury and demonstrates potential as a gene therapy intervention for combined radiation-trauma injury in clinic.

## INTRODUCTION

Radiation combined injury (RCI) is used to describe conditions in which radiation injury coincides with other injuries such as burns, skin wounds, infection or blunt trauma [1]. RCI is projected to account for up to 65% of all injuries following an atomic bomb event and it notably complicates the clinical prognoses of acute radiation sickness. RCI significantly increases the risk of morbidity and mortality compared to either type of injury alone, due to myelosuppression, immune system depletion, delayed traumatic wound healing, sepsis, multi-organ dysfunction syndromes and multi-organ failure [1, 2]. The National Institute of Allergy and Infectious Diseases has recognized RCI as a key issue [3]. One particular type of RCI, combined radiation-trauma skin injury, is characterized by a marked delay in wound healing, intensifying the psychological and financial burden on patients [4]. Studies have shown that the presence of wounds could shift the radiation survival curves for mice to the left, consequently reducing LD50 [5]. Therefore, the promoting wound healing becomes an attractive strategy for mitigating combined radiation-trauma skin injury. Although there currently exist numerous clinical treatments for combined radiation-trauma skin injury, for example, antioxidant therapy [6], recombinant epithelial cell growth factor [7] and recently developed stem cells [8], the efficacy of these treatment has not been satisfactory. There is a pressing need for the development of new and more effective medications and therapeutic strategies in clinical practice.

Oxidative stress and inflammation are the most important mechanisms of combined radiation-trauma skin injury to radiation, which can significantly delay wound healing processes [2]. Ionizing radiation damages vital intracellular bio-molecules resulting in multiple cellular and tissue injuries as well as pathophysiological responses such as inflammation, immunosuppression, etc. [9]. These damages increase flux of reactive oxygen species (ROS), disturb the normal redox homeostasis and lead to an ‘oxidative stress’ state. In this state, the intracellular ROS concentration surpasses the counteractive capacity of antioxidant defense mechanisms [9, 10]. In combined radiation-trauma skin injury, radiation-induced damage to wound repair cells (e.g. vascular endothelial cells, fibroblasts and epithelial cells) triggers a cascade of molecular events, including ROS production and DNA damage, which results in an early immune response aimed at initiating tissue repair [11]. Subsequently, proinflammatory mediators such as interleukin (IL)-1 and tumor necrosis factor-α (TNF-α) are released, initiating resident immune cells activation and inflammatory cells recruitment, which, in turn, amplifies the ongoing inflammatory response, eventually resulting in chronic inflammation and extensive radiation damage [12]. Our previous study found that the ratio of Th1/Th2 [Interferon-γ (IFN-γ)/IL-4] significantly increased in the combined wound and radiation injury group, causing disturbance of immunoregulation and delayed wound healing, and suggesting that proinflammatory Th1 responses affect the progression of the wound combined with local radiation injury [13]. The inflammatory process can induce oxidative stress and reduce cellular antioxidant capacity. Numerous studies have revealed that antioxidants not only have antioxidant activities, but also exert an effective role as anti-inflammatory factor [14]. Schaue *et al*. [15] reported that the antioxidative stress regulator Nrf2 suppressed radiation-induced NF-κB proinflammatory responses and switched Th1 responses to Th2 polarity. Thus, oxidative stress and chronic inflammation induced by ionizing radiation are crucially responsible for wound healing of combined radiation-trauma skin injury, and Nrf2 may be a key therapeutic target.

Hepatocyte growth factor (HGF), a pleiotropic growth factor, is well known to regulate cell proliferation, cell survival, angiogenesis, anti-inflammation and antioxidation [16–18]. Studies have demonstrated the ability of HGF to enhance the proliferation and migration of keratinocytes, promote capillary outgrowth and facilitate wound healing in diabetic mice [19]. However, the use of HGF in clinical practice is challenging due to its susceptibility to degradation *in vivo* [20]. So we constructed plasmid pHGF encoding the human HGF gene. Our previous studies showed that pHGF can induce angiogenesis on the chicken chorioallantoic membrane [21, 22]. Furthermore, intramuscular injection of pHGF in rat hindlimbs resulted in strong expression of HGF and a significant increase in capillary density in the ischemic tissue [23]. This treatment also significantly increased the blood flow in collateral vessels in either completely or partly femoral artery ligation hindlimb dog model [24]. In addition, no obvious toxic effects were observed in toxicity studies conducted in rats and dogs. Our Phases I and II clinical study indicated that intramuscular injection of plasmid pHGF in the limbs of patients with critical limb ischemia (CLI) was well tolerated, significantly reduced resting pain, and promoted better ulcer healing in CLI patients [25, 26]. Therefore, HGF not only exhibits antioxidant and anti-inflammation activity, but also promotes angiogenesis. This suggests that pHGF might have potential for gene therapy of refractory injury, such as the combined radiation-trauma skin injury. In this study, we investigated the effects of pHGF on protecting irradiated human immortalized keratinocytes (HaCaT) cells *in vitro*, as well as its ability to promote wound healing and antioxidation effects through local injection of pHGF on combined radiation-trauma skin injury in mice.

## MATERIALS AND METHODS

### Plasmid pHGF

**Fig. 1:**
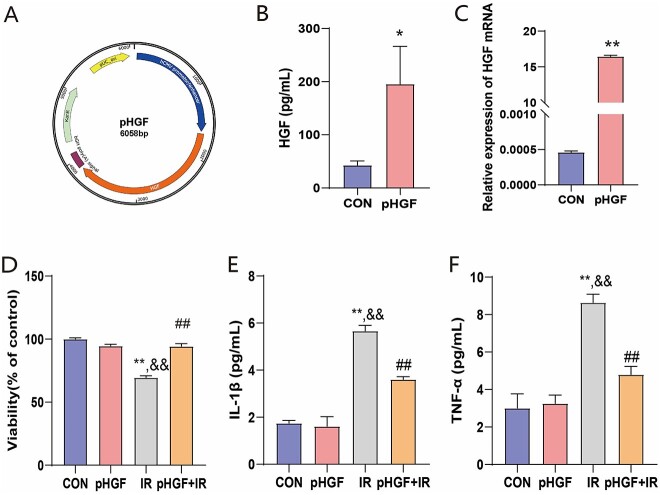
Effects of pHGF on cell viability and cytokine expression of irradiated HaCaT cells. (**A**) The plasmid profile of pHGF. (**B**) The protein expression of HGF in HaCaT cells post pHGF transfection measured by ELISA. (**C**) The mRNA expression of HGF in HaCaT cells post pHGF transfection assessed by qPCR. (**D**)The cell viability post different treatment detected by CCK-8. (**E**) The expression of IL-1β in the supernatant of HaCaT cells 24 h after irradiation. (**F**) The expression of TNF-α in the supernatant of HaCaT cells 24 h after irradiation. ^*^P < 0.05, ^**^P < 0.05 vs CON group; ^##^*P* < 0.01 vs IR group; ^&&^*P* < 0.05 vs pHGF group.

The plasmid pHGF, encoding human HGF gene, which is driven by the human cytomegalovirus promoter, was previously constructed and prepared in our laboratory. The plasmid map of pHGF is depicted in Figure 1A.

### Cell culture and transfection

Immortalized human epidermal cells (HaCaT cells) were cultured in DMEM/F12 (Gibco, USA) supplemented with 10% fetal bovine serum (FBS; Excell Bio, China) and penicillin–streptomycin (100 μg/ml penicillin and 100 μg/ml streptomycin, Solarbio, China) at 37°C in a 5% CO_2_ saturated humidified incubator.

HaCaT cells were seeded at a density of 3.5 × 10^5^ cells/well in 6-well plates, and transfection was performed when the cells reached 70% confluency. For transfection, 10 μl lipofectamine 2000 (11668027, Invitrogen, USA) and 4 μg pHGF were separately diluted in 250 μl Dulbecco's Modified Eagle Medium (DMEM)/F12 serum-free medium, then the two solutions were mixed, and incubated at room temperature for 20 min. The resulting 500 μl reaction mixture was added to the cells and gently shaken to ensure thorough mixing. After 6-h incubation, the media were replaced with fresh media containing 10% FBS.

### Cell irradiation

HaCaT cells were divided into four groups: control group (CON), irradiation group (IR), pHGF group (pHGF) and pHGF+irradiation group (pHGF+IR). The pHGF and pHGF+IR groups were transfected with pHGF. Cells in the IR and pHGF+IR groups were irradiated with 20 Gy X-rays (the dose rate 1.175 Gy/min, RS2000 X-ray irradiator, Rad Source Technologies, USA) at 24 h after transfection, while cells in the control and pHGF groups received sham irradiation simultaneously.

### Cell activity assay

HaCaT cells transfected with pHGF were seeded into 96-well plates at a density of 1.0 × 10^4^ cells/well and incubated overnight. Then 10 μl of CCK-8 solution (B34304, Bimake, USA) was added to the cells 24 h after irradiation, and the plates were incubated at 37°C for 2 h. The absorbance values were measured at 450 nm using the Multiskan MK3 enzyme-labeled instrument (Thermo Fisher Scientific, USA). Cell viability was calculated based on the absorbance values of the sample and the control group.

### Quantitative real-time polymerase chain reaction

After transfection and irradiation, the culture medium was discarded from the 6-well plate, and the cells were rinsed with ice-cold phosphate-buffered saline (PBS). Total RNA was extracted by adding 1 ml of TRIzol (15596026, Invitrogen, USA) to each well. The concentration and purity of RNA were assessed using a Nano-300 microspectrophotometer (ɅLLSHENG, China). Next, the RNA was reverse transcribed into cDNA using the One-Step gDNA Removal and cDNA Synthesis SuperMix (AT311-02, TransScript®, China). Quantitative real-time polymerase chain reaction was performed to quantify mRNA expressions of *Nrf2*, *Bcl-2* and *Bax*, with GAPDH serving as an internal reference, according to the instructions of PerfectStart® Green qPCR SuperMix (AQ601-01, TransScript®, China). Briefly, 10 μl PerfectStart Green qPCR SuperMix (2×), 0.4 μl Passive Reference Dye II (50×), 0.4 μl forward Primer, 0.4 μl reverse Primer, 1 μl cDNA and 7.8 μl ddH_2_O were added to the qPCR reaction strip tubes. The cycling conditions were as follows: denaturation at 95°C for 1 min, followed by 40 cycles of 95°C for 20 s, 60°C for 20 s and 72°C for 30 s. Fluorescence was determined on a QuantStudio 3 detection system (Applied Biosystems, Thermo Fisher Scientific, USA). The expression levels of *HGF, Nrf2*, *Bcl-2* and *Bax* between groups were statistically analyzed using the 2^−ΔΔCt^ method. The primer sequences are shown in Table 1.

**Table 1:** Primers for different genes

Gene	Primer sequence
*HGF*	F: GCTATCGGGGTAAAGACCTACA R: CGTAGCGTACCTCTGGATTGC
*Nrf2*	F: TGACAATGAGGTTTCTTCGG
	R: AGCAATGAAGACTGGGCTCT
*Bcl-2*	F: ATGTGTGTGGAGAGCGTCAACC
	R: TGAGCAGAGTCTTCAGAGACAGCC
*Bax*	F: TGGCAGCTGACATGTTTTCTGAC
	R: TCACCCAACCACCCTGGTCTT
*GAPDH*	F: CACTAGGCGCTCACTGTTCTC
	R: AAATCCGTTGACTCCGACCT

### Western blot

HaCaT cells were harvested with protein lysis buffer. And the protein concentrations were assessed using bicinchoninic acid (BCA) Protein Assay kit (Thermo Fisher Scientific, Waltham, MA). Protein samples were separated by 10% sodium dodecyl sulfate-polyacrylamide gel electrophoresis at 100 V for 2 h, and then transferred to nitrocellulose membranes. After blocking with 5% dried nonfat milk for 1 h at room temperature, the membranes were incubated with primary antibodies, such as anti-Nrf2 (1:1000, 16396-1-AP, Proteintech, China), anti-Bcl-2 (1:1000, A19693, Abclonal, China), anti-Bax (1:1000, A19684, Abclonal, China) or anti-β-actin (1:10 000, AC026, Abclonal, China), respectively, overnight at 4°C. Following three rinses, blots were incubated with horseradish peroxidase-conjugated secondary antibodies (1:10 000, SA0001-2, Proteintech, China) for 1 h at room temperature. Then the chemiluminescence signal in the blots was detected with Hypersensitive Chemiluminescence Substrate kit (17046, Zenbio, China) using the ePhoto developer (GenScript, China). The relative expression of the target protein was assessed using the internal reference β-actin as the standard. The intensity was quantified by ImageJ software (National Institutes of Health, Germany).

### Enzyme-linked immunosorbent assay

Supernatant collected from the 6-well plates was assayed in duplicate using either IL-1β (EK101B, Multi Sciences, China) or TNF-α enzyme*-*linked immunosorbent assay (ELISA) kits (EK182, Multi Sciences, China) according to the manufacturer’s instructions. Briefly，the standard substance and the test sample were added to the polyclonal antibody transparent enzyme-labeled plate. Then the enzymatic working solution was added and incubated for 2 h at room temperature. After washing four times, Substrates A and B were added, and the absorbance was measured at 450 and 630 nm. The concentrations of IL-1β and TNF-α were calculated according to the absorbance values of the standard substance and test samples.

### Animals

A total of 125 SPF grade C57BL/6 J male mice (6–8 weeks) were purchased from SPF (Beijing) Biotechnology Co., Ltd (Beijing, China). The mice were kept with standard conditions under a 12-h light–dark cycle, with access to food and water ad libitum. The animal welfare and operation procedure were strictly adhered to the Laboratory Animals Guideline of welfare and ethics (GB/T 35892-2018), and were approved by the animal care and use committee at Beijing Institute of Radiation Medicines.

### Skin injury in mice and experimental protocol

The combined radiation-trauma skin injury mice model was implemented according to the previously published methods [13]. Mice were randomly divided into the following groups according to the body weight (25 mice per group): wound group, wound and RCI group, pHGF low-dose treatment group (L-HGF group, 50 μg/cm^2^), pHGF medium-dose treatment group (M-HGF group, 100 μg/cm^2^) and pHGF high-dose treatment group (H-HGF group, 150 μg/cm^2^). Mice were anesthetized with 1% sodium pentobarbital i.p. Post-shaving the dorsal surface, a full-thickness skin wound (1.3 cm in diameter) was created on each mouse’s back. Mice were then fixed in a special device, and a total body irradiation of 6 Gy was administered using the ^60^Coγ-ray source at a dose rate of 66.7 cGy/min. After irradiation, pHGF was administered via multi-point injection around the wound one-time only. The RCI group received an equal volume of PBS.

### General observation and wound area measurement

Graphics of dorsal wounds (*n* = 4–5/group) were taken and quatified using Image-Pro Plus 6.0 (Media Cybernetics, USA) on Days 1, 3, 7, 14 and 21 postirradiation. The area measured on Day 1 after treatment was regarded as the initial area, and the relative remaining area was calculated in the subsequent days (Relative wound area ratio = wound area/initial area×100%).

### Cytokine assay

Peripheral blood collected from mice on Days 1, 3, 7 and 14 post treatment was centrifuged at 3000 rpm for 10 min to separate the serum. The expression of various cytokines (IL-6, IL-4, IFN-γ and TNF-α) in serum was measured using the LEGEND plex™ MU Th1/Th2 panel (8-plex) (741 054, Biolegend, USA). The assay was performed according to the instructions. Briefly, samples and mixing beads were transferred into 96-well plates and incubated for 2 h with shaking in the dark. After washing, 25 μl of detection antibodies were added into each well and incubated for 1 h with shaking in the dark. Subsequently, 25 μl of SA-PE was added to each well and incubated for 30 min with shaking in the dark. Then the beads were washed twice and resuspended in wash Buffer. Cytokines detection was performed using BD LSR Fortessa^TM^ flow cytometer (BD, NJ), and data were analyzed on legendplex.qognit.

### Pathological observation

Tissues were fixed in 10% formalin solution and embedded in paraffin and then sectioned. The sections (4 μm) were stained with hematoxylin and eosin and observed under optical microscope (DM750, Leica, Germany) for histological analysis.

### Immunohistochemistry

Skin tissue sections were deparaffinized, rehydrated and blocked with 3% H_2_O_2_ for 15 min. The sections were incubated with primary antibodies such as anti-HGF (1:1200, DF6326, Affinty Biosciences, China), anti-Nrf2 (1:3000, 16 396–1-AP, Proteintech, China), anti-Bcl-2 (1:100, A19693, Abclonal, China), anti-Bax (1:5000, A19684, Abclonal, China) or anti-vascular endothelial growth factor (VEGF; 1:7000, 19 003–1-AP, Proteintech, China) at 4°C overnight. After thorough washing, sections were incubated with horseradish peroxidase-conjugated secondary antibody (1:5000, Proteintech, China) for 30 min at room temperature, then stained with diaminobenzidine solution. Sections were visualized under a microscope, and representative sections were scanned and analysed using a biopsy scanner (NDP, Hamamatsu, Japan). ImageJ software quantified the percentage contribution of the positive with four random images were taken from each group.

### Apoptosis analysis by TUNEL

Apoptotic cells in the wound region were labelled via the terminal deoxynucleotidyl transferase dUTP nick end labelling (TUNEL) method according to the instructions of Colorimetric TUNEL Apoptosis Assay Kit (C1098, Beyotime, China). Cells that exhibited brownish-yellow particles within the nucleus were considered positive. Positive cells were counted by selecting four complete and nonoverlapping microscopic fields (400× magnification).

### Statistics

Normally distributed continuous variables were expressed as mean ± SD. The data were analyzed using Graphpad Prism 8.0 (Graphpad, Boston, MA). Differences were analyzed by using one-way Analysis of Variance (ANOVA) followed by Fisher least significant differences test to determine differences among groups. *P* < 0.05 was considered to be statistically significant.

## RESULTS

### pHGF protected the irradiated HaCaT cells via inhibition of inflammation and apoptosis

To detect the protective effect of pHGF on radiated HaCaT cells, pHGF was transfected into HaCaT cells. The protein and mRNA expressions increased markedly post transfection (Fig. 1B and C). Following exposure to 20 Gy X-ray irradiation, HaCaT cell viability in IR group significantly decreased compared with the nonirradiated CON group (*P* < 0.001). On the contrary, pHGF transfection administered 24 h prior to irradiation significantly increased the cell viability in irradiated HaCaT cells (*P* < 0.01) and achieved the level observed under normal culture condition (Fig. 1D).

To investigate the effect of pHGF on the secretion of inflammatory cytokines post irradiation, cell supernatants were collected at 24 h after irradiation. The results showed that the levels of IL-1β and TNF-α in the supernatant were significantly higher in IR group compared with the CON group (*P* < 0.001). However, the secretion of IL-1β and TNF-α in irradiated HaCaT cells was significantly inhibited in pHGF+IR group compared with that in IR group (*P* < 0.01, Fig. 1E and F).

**Fig. 2:**
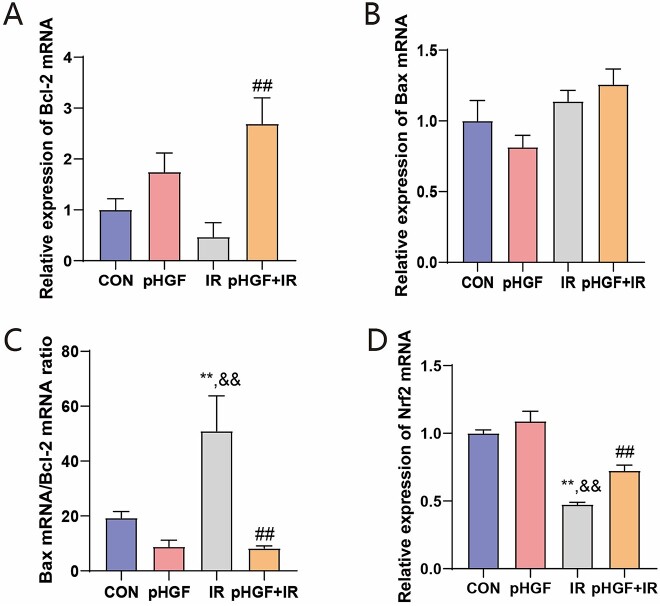
Effects of pHGF on the mRNA expression of Bcl-2, Bax and Nrf2 in irradiated HaCaT cells. mRNA expression of Bcl-2 (**A**), Bax (**B**) and Nrf2 (**D**) was detected by RT-qPCR. (**C**) The ratio of Bax mRNA/Bcl-2 mRNA. ^**^P < 0.01 vs CON group, ^##^*P* < 0.01 vs IR group, ^&&^*P* < 0.01 vs pHGF group.

**Fig. 3:**
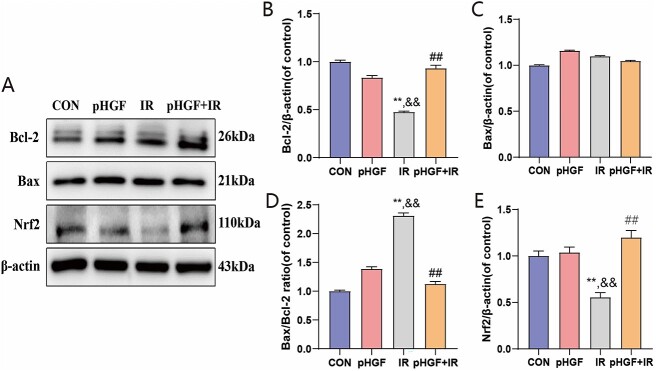
Effects of pHGF on the protein expressions of Nrf2, Bax and Bcl-2 in irradiated HaCaT cells. (**A**) The protein expression of Bcl-2, Bax and Nrf2 in HaCaT cells with different treatments was detected by western blot. (**B**) The relative proteins expression of Bcl-2. (**C**) The relative proteins expression of Bax. (**D**) The ratio of Bax protein/Bcl-2 protein. (**E**) The relative proteins expression of Nrf2. ^**^P < 0.01 vs CON group, ^##^*P* < 0.01 vs IR group, ^&&^*P* < 0.01 vs pHGF group.

Furthermore, we evaluated the mRNA expression levels of the antiapoptotic protein Bcl-2 and proapoptosis protein Bax after pHGF treatment in irradiated HaCaT cells through. As shown in Figure 2, Bcl-2 expression was downregulated (Fig. 2A) and Bax expression was upregulated (Fig. 2B) in the irradiated HaCaT cells, and the Bax/Bcl-2 ratio was significantly increased compared to the CON group (*P* < 0.01, Fig. 2C). Meanwhile, the pHGF+IR group demonstrated a significant upregulation of Bcl-2 (*P* < 0.001) and a decrease in the Bax/Bcl-2 ratio (*P* < 0.001) compared with the IR group. Consistently, the tendency of the protein expressions of Bax and Bcl-2 was similar with the mRNA expression, the protein expression of Bcl-2 was significantly upregulated in the pHGF+IR group and a corresponding significant reduction in the Bax/Bcl-2 ratio (Fig. 3).

### pHGF regulated Nrf2 pathway

As Nrf2 is a key regulator for antioxidation, cell apoptosis and inflammatory response subjected to ionizing radiation, we investigated whether pHGF might regulate Nrf2 expression in irradiated HaCaT cells. After irradiation, both protein and mRNA expression of Nrf2 demonstrated significant downregulation in irradiated HaCaT cells (IR group) compared with the CON group (Figs 2D and 3E, *P* < 0.001). However, pHGF transfection upregulated Nrf2 mRNA expression in irradiated HaCaT cells compared with the IR group (*P* < 0.01). Similarly, the protein expression of Nrf2 in pHGF+IR group increased significantly compared with the IR group (*P* < 0.001).

### Therapeutic effects of pHGF against radiation-combined injuries in mice

**Fig. 4:**
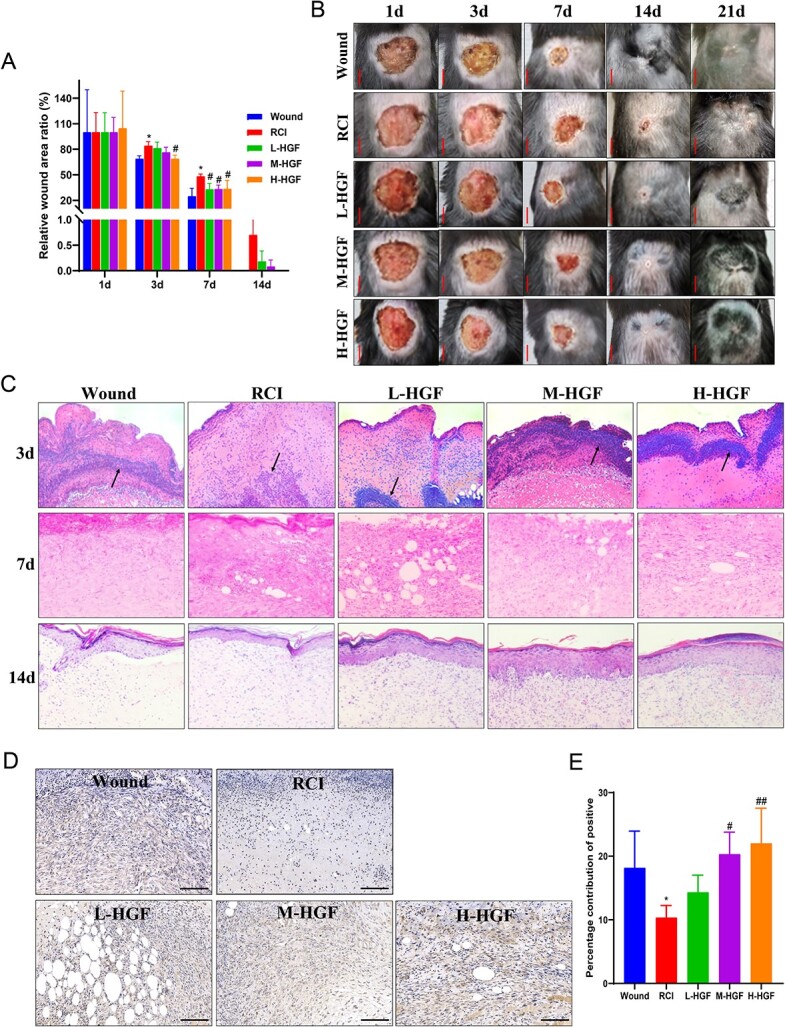
Effects of pHGF on wound healing in mice with combined radiation-trauma injury. (**A**) On Days 1, 3, 7, 14 and 21 after injury, the wound healing graphics were taken. Scale bar = 5 mm. (**B**) The relative wound area ratio in different groups after injury. (**C**) Hematoxylin and eosin staining of the wound tissue on Days 3, 7 and 14 in different groups. Black arrow: inflammatory cells. Scale bar = 100 μm. (**D**) HGF expression in the wounds of mice in various groups at Days 7 detected by immunohistochemistry staining. Scale bar = 100 μm. Representative results were given. (**E**) Percentage contribution of the positive HGF expression. ^*^P < 0.05 vs Wound group; ^#^*P* < 0.05, ^##^*P* < 0.01 vs RCI group.

As shown in Figure 4A and B, the wound combined with a single dose of 6 Gy γ-ray whole body radiation led to obvious delay in wound healing. On Days 3 and 7 after injury and irradiation, the relative wound area ratio of the RCI group (~84.1 and 47.7% respectively) was significantly larger than that of the wound group (~68.7 and 24.7%, respectively) (*P* < 0.05).

After local administration of pHGF, wound healing improved from Day 3 to 14. On Day 3, only the H-HGF group (relative wound area ratio was ~68.7%) showed a significant reduction in relative wound area ratio compared with that of the RCI group. On Day 7, wounds in all pHGF treatment groups demonstrated faster healing than the RCI group, and the relative wound area ratio was ~32.9% in L-HGF, 33.2% in M-HGF and 33.3% in H-HGF, respectively, which reduced by ~14.8, 14.5 and 14.4%, compared to the RCI group (*P* < 0.05). On Day 14, wounds in all pHGF treatment groups had fully healed, 7 days earlier before the wounds in the RCI group (Fig. 4B).

Histological observation showed marked improvement in pHGF-treated wounds: obvious wound exudate and a notable ‘isolation band’ of inflammatory cells on Day 3, abundant granulation tissue and increased neovascularization on Day 7 and full coverage of the wounds by epidermal cells with a formed stratum corneum in all pHGF-treated groups on Day 14 (Fig. 4C).

Immunohistochemistry results showed increased expression of HGF protein in M-HGF and H-HGF-treated skin tissues on Day 7 postirradiation (Fig. 4D and E, *P* < 0.05), indicating that a single injection of pHGF locally can induce the expression of HGF protein in skin tissues for up to 7 days.

**Fig. 5:**
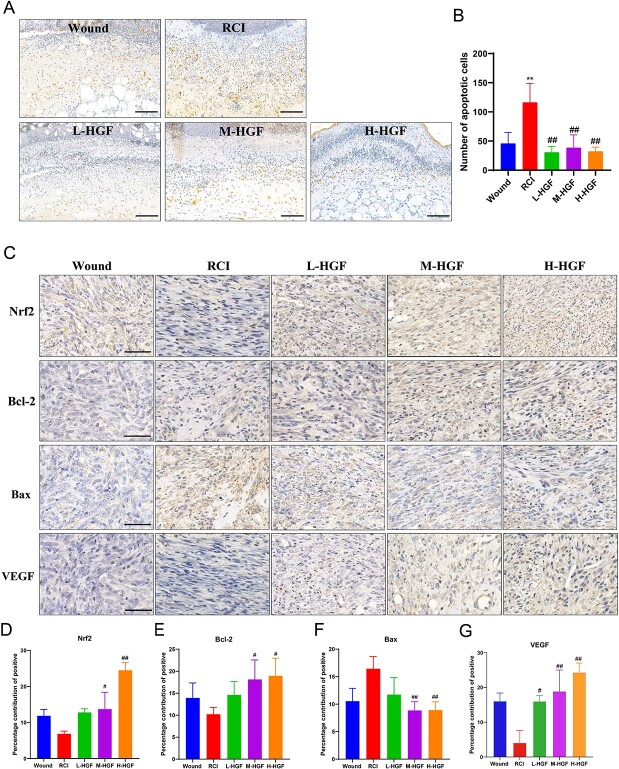
Effects of pHGF on the apoptosis and Nrf2, Bcl-2, Bax and VEGF expression in wounds of mice with combined radiation-trauma injury. (**A**) On Day 3 after injury, the apoptosis in wounds in different groups was detected by TUNEL. Scale bar = 100 μm. (**B**) The average positive apoptotic cells in the wounds of mice in various groups. (**C**) On Day 7 after injury, the expression levels of Nrf2, Bcl-2, Bax and VEGF were evaluated by IHC. Scale bar = 50 μm. Representative results were given. (**D–G**) Percentage contribution of the positive Nrf2, Bcl-2, Bax and VEGF expression. ^**^P < 0.01, vs wound group; ^#^*P* < 0.05, ^##^*P* < 0.01 vs RCI group.

Findings from the TUNEL assay showed that pHGF inhibited apoptosis induced by combined radiation-trauma injuries (Fig. 5A). The number of apoptotic cells in the wounds of RCI group mice increased significantly on Day 3 compared to that of the wound-alone group (Fig. 5B, *P* < 0.01). Whereas the number of apoptotic cells reduced significantly in all three pHGF treatment groups compared with the RCI group mice (*P* < 0.01).

Furthermore, we measured the expressions of Nrf2 and apoptotic-related proteins, Bcl-2 (an apoptosis inhibitor) and Bax (an apoptosis promoter) in local wound tissue through immunohistochemistry. Results showed that Nrf2 and Bcl-2 expressions in the M-HGF and H-HGF groups displayed significantly higher than those in the RCI group on Day 7 (Fig. 5D and E). Meanwhile, the expression of Bax reduced significantly in these two groups (Fig. 5F).

Further analysis showed that pHGF upregulated the expression of VEGF gene, which is related to angiogenesis. VEGF expression in the skin tissues of all pHGF treatment groups was significantly higher than that in the RCI group on Day 7 (Fig. 5C and G).

**Fig. 6:**
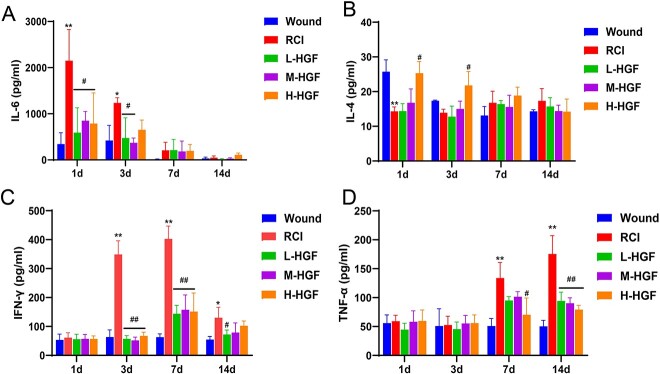
Cytokines in serum of mice in each group after injury. (**A**) IL-6. (**B**) IL-4. (**C**) Interferon-γ (IFN-γ). (D) TNF-α. ^*^P < 0.05, ^**^P < 0.01 vs wound group; ^#^*P* < 0.05, ^##^*P* < 0.01 vs RCI group.

The prolonged inflammatory response and impaired immune function caused by ionizing irradiation contribute to delayed wound recovery [11]. Thus, we examined the Th1/Th2 cytokines in serum and found that the levels of several cytokines significantly changed after radiation and wound. The content of IL-6 was significantly increased in the RCI group on Days 1 and 3 compared with wound group (Fig. 6A, *P* < 0.01). The level of IL-4 reduced significantly on Day 1 (Fig. 6B, *P* < 0.01), while the expressions of IFN-γ and TNF-α in the RCI group increased significantly on Days 3, 7 and 14 (Fig. 6C and D, *P* < 0.05). However, administration of pHGF significantly reduced IL-6 levels in L-HGF and M-HGF groups (Fig. 6A, *P* < 0.05), but enhanced IL-4 expression in the H-HGF group on Days 1 and 3 (Fig. 6B, *P* < 0.05). The levels of IFN-γ and TNF-α in pHGF treatment groups significantly decreased compared to the RCI group on Days 7 and 14 (Fig. 6C and D, *P* < 0.01), respectively.

## DISCUSSION

Wound healing is a complex, intricately coordinated process involving overlapping phases of inflammation, proliferation and remodeling. However, the healing of combined radiation-trauma skin injury becomes substantially complex due to the disruptive effects of ionizing radiation [27]. Due to the combined effects, combined radiation-trauma skin injury diverges significantly from singular wound or radiation injury, and the healing of wounds is far more complicated [28]. On a molecular level, ionizing radiation induces DNA double-strand breaks and amplifies intracellular ROS accumulation. ROS plays an important regulatory role in several stages of wound healing, low levels of ROS are required for hemostasis, lymphocyte recruitment, pathogen defense and tissue repair [29]. However, an excessive influx of ROS can instigate peroxidation reaction, escalate the secretion of inflammatory cytokines and ultimately lead to cell death, by assaulting proteins, fatty acids, nucleotides and other molecules [30, 31]. At the tissue level, persistent inflammation, reduced angiogenesis, diminished cell growth factors and collagen synthesis, along with an excessive degradation of extracellular matrix, collectively impede repithelialization and delay the wound healing of combined radiation-trauma skin injuries [3]. A previous study showed that HGF efficiently downregulated ROS generation and oxidative damage, which was related to an increment in glutathione (GSH) synthesis. Meanwhile, HGF activates canonical survival pathways such as Akt and Erk1/2, which are related to Nrf2 activation, and the increment of γ-glutamyl cysteine synthetase (γ-GCS), a main canonical target of Nrf2 [32].

Nrf2 is a member of the family of basic leucine zipper transcription factors, originally identified as a key regulator of canonical antioxidant and drug metabolizing gene expression [33, 34]. Under oxidative stress, Nrf2 is released from Keap1 and translocates to the nucleus where it upregulates ARE-dependent cytoprotective genes such as glutathione transferases, UDP-glucuronosyltransferases, γ-GCS, GSH peroxidase, heme oxygenase-1 (HO-1), catalase and NAD(P)H:quinone oxidoreductase-1 [34]. Several researches identified Nrf2 as a critical factor for promoting survival of mammalian cells subjected to ionizing radiation, through ROS detoxification, DNA repair facilitation and potential modulation of anti-inflammatory cytokine responses [35]. Rhyu *et al*. [36] reported that ROS generation is linked to the epithelial-to-mesenchymal transition process by TGFβ1 in rat renal epithelial cells and demonstrated that the inhibition of ROS generation could reduce the TGFβ1-stimulated fibronectin secretion. It is also noteworthy that the Bcl-2 family proteins, comprising antiapoptotic members including Bcl-2 and numerous proapoptotic members, regulate cell death and survival. Niture *et al*. [37] demonstrated that Nrf2 enhances antiapoptotic Bcl-2 expression, while suppressing proapoptotic Bax expression to directly inhibit cell apoptosis. Loss of Nrf2 leads to increased accumulation of ROS and renders cell and mice radiosensitive [34, 38]. Taken together, the Nrf2 antioxidant could be suggested as an effective target to enhance combined radiation-trauma skin injury wound healing.

In our study, we found that pHGF transfection prior to irradiation resulted in an upregulation of Nrf2, Blc-2, downregulation of Bax and inhibition of inflammatory cytokines such as IL-1β and TNF-α in irradiated HaCaT cells *in vitro*, indicating that the radioprotective effect of pHGF was related to the activation of Nrf2 pathway and inhibition of inflammatory and apoptosis.

*In vivo* studies, we designed a protocol of subcutaneous injection of naked plasmid pHGF to investigate its therapeutic effects on combined radiation-trauma in mice. A single injection of pHGF into the irradiated skin area was enough to promote HGF protein expression that lasted for 7 days. pHGF administration mitigated the progress of skin injury and significantly enhanced the wound healing, and the wounds in the pHGF-treated groups appeared to be normalized on Day 14 postirradiation. Additionally, pHGF was found to enhance Nrf2 and Bcl-2 protein expression, inhibit Bax protein expression and decrease apoptosis in local wound tissue. It is reported that HGF can induce an Nrf2-mediated antioxidant protective response through inducing NADPH oxidase-dependent protection against the toxicity elicited by Antimycin A, an inhibitor of the respiratory chain [39]. Al-Sawaf *et al*. also discovered that HGF can induce Nrf2 activity in myoblasts [40]. Combined with the results, we supposed that regulating theNrf2 pathway is related to pHGF’s radioprotective effects.

Radiation-induced endothelial/vascular injury is another major complicating factor impeding the recovery from combined radiation-trauma skin injury. Ionizing radiation generates oxidative stress, causing microvascular endothelial cells damage [5]. VEGF, which plays an important role in the regulation of angiogenesis, has been found to interact with Nrf2 [41]. Kuang *et al*. [42] found that hypoxia upregulates the mRNA and protein expression of Nrf2, HO-1 and VEGF, with a concomitant increase in cell migration and vascular tube formation. However, knockdown of Nrf2 markedly decreased HO-1 and VEGF expression. Furthermore, Li *et al*. [43] indicated that Nrf2 regulates angiogenesis via HO-1-mediated HIF-1α/VEGF signaling pathways. In our study, we found that pHGF enhanced the expression of VEGF protein in the wound skin tissues, suggesting that the pHGF might induce VEGF expression and regulate angiogenesis through the activation of the Nrf2 pathway.

It is known that immune system inhibition by combined radiation-trauma skin injury is the main component of chronic inflammation [2]. Th1 and Th2 cells are critical for the pro- and anti-inflammatory immune modulation. Th1 cells produce IFN-γ and TNF-α, activate macrophages and promote cell-mediated immunity, while IFN-γ aids in the polarization of naive T cells to Th1 cells. In contrast, Th2 cells mainly produce IL-4, which is a key factor in Th2 cell differentiation, B cell proliferation and immunoglobulin class switching [44, 45]. Therefore, the levels of IFN-γ and IL-4 could reflect immune balance of Th1/Th2. Consistent with our previous study [13], this study demonstrated that IL-4 level in the RCI group significantly decreased on Day 1, while the IFN-γ and TNF-α levels in the RCI group remained significantly increased on Day 14. This indicates that the Th1/Th2 balance is impaired, shifting toward a more proinflammatory Th1. After pHGF administration, IL-4 level in H-HGF group significantly increased on Days 1 and 3, while IFN-γ levels in all pHGF treatment groups significantly decreased on Days 3 and 7. This indicates that pHGF could inhibit inflammatory reaction, which was related to wound healing.

All these results suggest that regulating the Nrf2 pathway is part of the mechanisms responsible for pHGF promoting the healing of combined radiation-trauma skin injury. Further investigations are required to establish this relationship in detail.

## CONCLUSIONS

In summary, this study provides evidences that the protection conferred by pHGF on irradiated cells could be associated with the regulation of the Nrf2 pathway. The simple multi-site injections of naked plasmid pHGF exhibit a noticeable therapeutic effect on combined radiation-trauma skin injury in mice. These findings suggest that pHGF has potential for gene therapy in the treatment of impaired wound healing, such as combined radiation-trauma skin injury.

## CONFLICT OF INTEREST

We declare that we have no conflict of interest.

## Funding

The work to produce this paper did not receive any specific grant.
